# Impact of immunodeficiencies on immunity induced by SARS-CoV-2 infection, mRNA BNT162b2 vaccination, and their combination in children and young adults

**DOI:** 10.3389/fimmu.2025.1661282

**Published:** 2025-11-07

**Authors:** Lubica Fialova, Birivan Macek-Nabova, Monika Zilkova, Natalia Turic-Csokova, Denisa Palova, Stanislav Katina, Gabriela Paulikova-Rolkova, Peter Ciznar, Julia Horakova, Lubica Wojciakova, Karina Markova, Eva Kontsekova, Branislav Kovacech

**Affiliations:** 1 Institute of Neuroimmunology, Slovak Academy of Sciences, Bratislava, Slovakia; 2 AXON Neuroscience R&D Services SE, Bratislava, Slovakia; 3 Department of Paediatric, National Institute of Children’s Diseases, Bratislava, Slovakia; 4 Faculty of Science, Institute of Mathematics and Statistics, Masaryk University, Brno, Czechia; 5 Department of Paediatric, Medical Faculty Comenius University in Bratislava, Bratislava, Slovakia; 6 Department of Paediatric Haematology and Oncology, National Institute of Children’s Diseases, Bratislava, Slovakia

**Keywords:** SARS-CoV-2, primary and secondary immunodeficiencies, mRNA BNT162b2 vaccine, inhibitory antibodies, CD4 T-cells, Geometric mean titre

## Abstract

Current understanding of how immunodeficiencies impact protective responses against viral infections and vaccination is primarily derived from adult cohorts that may not accurately reflect the pediatric, adolescent, and young adult population. This cross-sectional study aimed to evaluate immune responses in this underrepresented population affected by various immunodeficiencies after SARS-CoV-2 infection, two doses of the mRNA BNT162b2 vaccine, or after a combination of both. We analyzed blood samples from 102 immunocompromised patients (IC) (5–25 years) categorized into groups of primary immunodeficiencies (PID, n=17), bronchial asthma and allergic rhinitis (BA-AR, n=39), rheumatoid diseases (RD, n=21), and individuals who had undergone hematopoietic stem cell transplantation (HSCT, n=28), as well as 30 healthy individuals (9–26 years). We measured titres of Spike-specific IgM, IgA, and IgG antibody classes (including IgG subclasses) in plasma using ELISA and evaluated their inhibitory potential in a Spike-ACE2 cell-based internalization assay. Spike-specific CD4 T-cells were examined using a flow cytometry-based proliferation assay (FASCIA). In the IC group, all participants except eight generated detectable levels of IgG antibodies. The IgG titres induced by vaccination (Geometric mean titre (GMT_vac_) = 205023, 95% CI: 116074-362136) and a combination of vaccination and infection (GMT_hyb_ = 172819, 95% CI: 33133-901403) were higher than after infection (GMT_inf_ = 3323, 95% CI: 578-19109, P_vac/inf_ = .006 and P_hyb/inf_ = .001). On the other hand, the hybrid immunity induced the highest IgA titres (GMT_hyb_ = 2672, 95% CI: 566-12623) compared to vaccination (GMP_vac_ = 275, 95% CI: 97-777, P_hyb/vac_ = .016) and infection (GMT_inf_ = 60, 95% CI: 13-280, P_hyb/inf_ = .002). The IgG titres in vaccinated and hybrid immunity groups strongly correlated (r_Spearman_ = 0.86, P <.0001) with the levels of antibodies inhibiting the internalization of Spike protein (S protein) in a cell-based assay. Most IC patients (except five) also developed above-threshold Spike-specific CD4 T-cell responses, which were not statistically different from the responses in the healthy control group. Our data show that infection and vaccination can induce protective humoral or cellular responses against SARS-CoV-2 in IC patients. The activated cellular response in patients with agammaglobulinemia may assist them in overcoming viral infections.

## 1 Introduction

Exposure to new antigens in immunocompromised (IC) populations, such as the situation that arose during the COVID-19 pandemic, provided a unique opportunity to evaluate the ability of patients’ immune systems to elicit functional antibodies and generate targeted cellular responses. This heterogeneous group of patients, comprising individuals with primary immunodeficiencies (PID, commonly referred to as inborn errors of immunity) and those with secondary immunodeficiencies resulting from chronic illnesses or their treatments, is actually a spectrum of rare diseases that share some common characteristics (1). PID comprise a highly varied group of disorders, with over 500 types recently recognized, exhibiting symptoms that differ in severity and in the immune pathways affected (2). While individual PID often have relatively narrow spectrum mechanisms, secondary immunodeficiencies typically impact multiple immune pathways.

Recent research has significantly enhanced our understanding of the host immune responses triggered by natural SARS-CoV-2 infection and by COVID-19 vaccines. Numerous studies have shown that SARS-CoV-2 vaccines stimulate both arms of the adaptive immune response and reduce the severity of COVID-19 disease in immunocompetent adults (3–7). However, several studies indicated a suboptimal immune response in immunocompromised adults (8–12). Our current understanding of how immunodeficiencies influence immune response against infections and vaccinations is primarily derived from studies conducted with adult populations. This may not accurately reflect the immune responses in pediatric and young adults, since some investigations indicate that immune response profiles to SARS-CoV-2 are distinct in children compared to adults (13–15). Therefore, more information is required to better understand the specific immune responses following vaccination and infection in IC children with rare diseases and to improve pediatric clinical practice.

In this study, we included a group of 102 children and adolescents with primary and secondary immunodeficiencies, along with 30 age-matched controls.

Our study offers a detailed profile of immune responses across various immunodeficiencies following vaccination, after SARS-CoV-2 infection, and as a result of a combination of both vaccination and infection. We measured total S-specific IgG, IgA, and IgM titres and investigated the different subclasses of IgG (IgG1, IgG2, IgG3, and IgG4) using ELISA. Additionally, we evaluated the inhibitory capacity of antibodies in plasma samples from all participants using cell-based assay. Moreover, we assessed the CD4 T-cell response triggered by the vaccine, by SARS-CoV-2 infection, and by both. Obtained data are crucial for understanding the immune system’s capabilities in individuals with deficiencies, providing valuable insights for clinicians treating young patients with immune defects.

## 2 Methods

### 2.1 Study design and sample collection

Children, adolescents, and young adults diagnosed with immunodeficiency syndromes, who were regularly monitored by the National Institute of Children’s Diseases in Bratislava, participated in the cross-sectional study running from July 2021 to May 2022. The inclusion criteria for study participation required either SARS-CoV-2 vaccination (Pfizer mRNA BNT162b2 vaccine) and/or SARS-CoV-2 infection confirmed by RT-PCR. The cohort consisted of 102 patients, aged between 5 and 25 years, who were suffering from primary immunodeficiencies (PID) and secondary immunodeficiencies. The PID group comprised patients with predominant antibody deficiencies, as well as patients with common variable immunodeficiency (CVID), individuals with severe T lymphocyte deficiencies, and those with newly described syndromic defects (22). The patients with secondary immunodeficiencies were categorized into three groups: the BA-AR group included patients with bronchial asthma and uncomplicated allergic rhinitis; the RD group comprised patients with rheumatoid diseases, primarily juvenile idiopathic arthritis; and the HSCT group consisted of patients who underwent hematopoietic stem cell transplantation. A control group was recruited from healthy children, adolescents and young adults to match the age range of the immunocompromised participants (9–26 years). The study received approval from the Ethics Committee of the National Institute of Children’s Diseases in Slovakia (EK6/2021), as well as from the Ethics Committee of the self-governing region of Bratislava (number 04848/2021/HF for the control group). Informed consent for participation was obtained from all subjects or their legal guardians, and a sample of heparinized whole blood was collected from patients during a routine health examination. Peripheral blood cells were used to determine T-cell responses, and plasma was used to assess the antibody response.

### 2.2 Preparation of cell line and Spike protein

Human embryonic kidney HEK293T/17-hACE2 cells with stable expression of human angiotensin-converting enzyme ACE2 (AXON Neuroscience SE) and recombinant S protein were prepared as described previously (16).

### 2.3 Determination of S-specific antibodies by ELISA

Recombinant S protein (100 ng/well in PBS) was immobilized on microtitre plates (High Binding plates; Greiner Bio-One, Germany) at 37°C for 2h. After blocking with PBS-0.1% Tween 20, the plates were incubated overnight with serially diluted patient plasma samples. After washing (PBS-0.1% Tween 20), bound antibodies were detected by anti-human immunoglobulins conjugated to HRP (anti-human class-specific secondary antibodies for the detection of IgM, IgG, IgA, and IgG subclasses IgG1, IgG2, IgG3, and IgG4, all from Thermo Fisher Scientific, USA). The bound secondary antibodies were measured through the HRP activity with the chromogenic substrate TMB One (Kementec Solutions A/S, Denmark) at the absorbance of 450 nm. The resulting signal was compared with that obtained for the negative human plasma collected before the COVID-19 pandemic (Pooled Human plasma K_3_EDTA, #15922, Innovative Research, Inc.). The titre of the antibodies in the plasma was defined as the highest dilution at which the absorbance at 450 nm was at least twice the absorbance of an equally diluted negative plasma sample. To ensure assay consistency and quality, positive control samples at three dilutions (for IgG: QC1 – 75,000x; QC2 – 10,000x; QC3 – 1,000x; for IgM: 20,000x; 3,000x; 500x; for IgA: 8,000x; 2,000x; 200x) of the plasma pool from 9 subjects with PCR positive SARS-CoV-2 to each plate were added. Negative sample plasma was serially diluted like patient plasma samples on each plate.

### 2.4 S-ACE2 binding inhibition assay

HEK 293T/17-hACE2 cells stably expressing human ACE2 protein (16), were seeded at 60-70% plating density in a 48-well plate and cultivated O/N at 37°C, 5% CO_2_ in DMEM supplemented with 10% (v/v) fetal bovine serum, 2 mM L-glutamine (all from GIBCO), gentamicin (0.05 mg/ml, Sigma-Aldrich), and 100 µg/ml hygromycin (Thermo Fisher Scientific). Recombinant Spike protein was labelled with Alexa Fluor™546 (Thermo Fisher Scientific) according to the manufacturer’s recommendations. 40 ng/ml of labelled S protein was pre-incubated with three-fold serially diluted plasma sample (from 100x to 24,300x) for 30 min at 37°C. Then the pre-incubated mixtures were added to HEK 293T/17-hACE2 cells and incubated for 2 hrs at 37°C, 5% CO_2_ in a humidified incubator. Subsequently, cultivation media were removed, and cells were gently resuspended in 500 µl of PBS and immediately evaluated for S protein internalization by flow cytometry (BD LSR Fortessa™, BD Bioscience). Measurements were recorded as mean fluorescent intensity of Alexa Fluor 546. 10,000 single cells were quantified for each plate well. Fluorescence of all tested samples was normalized by expressing it as percentage (sample%) of average fluorescence of wells with 100x diluted negative human plasma Pooled Human Plasma K3EDTA, Inc. (100%). The percentage of S protein uptake inhibition for all plasma samples was calculated by formula (inhibition% = 100 - sample%). The titre was determined as the highest dilution of plasma sample with inhibition effect (>= 20%) on S protein uptake. A positive control, consisting of a defined plasma pool of nine SARS-CoV-2 positive subjects verified by PCR, were added to each plate.

### 2.5 Determination of CD4 T-cell response

For determination of CD4 T-cells, the FASCIA assay was used (17, 18). Heparinized blood from donors was diluted 1:10 in RPMI 1640 culture medium (GIBCO) supplemented with L-glutamine (2 mM, GIBCO) and gentamicin (0.05 mg/ml, Sigma-Aldrich). The diluted blood sample was transferred into three sterile tubes in a volume of 500 µl/tube for stimulation with positive, negative and test stimuli. One tube (positive control) was stimulated with Concanavalin A (ConA, Sigma-Aldrich) at a final concentration of 10 µg/ml, another tube was stimulated with S protein at a final concentration of 100 µg/ml. The third tube remained unstimulated and served as a negative control (only diluted blood). The FACS tubes were incubated for 7 days at 37°C, 5% CO2 and 95% relative humidity. After incubation, the cell supernatant was removed and blood cells were stained with a mix of antibodies against activated T-cell surface markers (CD3-PECy7, CD4-PE, CD8-APC, all BD Biosciences) for 30 min at room temperature in the dark. Subsequently, erythrocytes were lysed by adding 1.5 ml of 1x lysing buffer/tube (BD Biosciences) for 10 min in the dark. After removal of the buffer by centrifugation (10 min at 350 g), the cells were washed with PBS, centrifuged for 10 min at 350 g and then the supernatant was decanted. Finally, cell pellets were resuspended in 450 μl of PBS and the blast numbers were immediately counted by flow cytometry (LSR Fortessa™, BD Biosciences) for 60 seconds. Unstimulated and ConA stimulated controls were applied for gate setting for resting lymphocytes and blasts (blasts were identified by their FSc/SSc properties as larger than resting and dying lymphocytes). CD3+ cells were divided into CD4+ cells (helper T-cells) and CD8+ cells (cytotoxic T-cells) on separate dot plots and gated. The number of CD4+ blasts in stimulated and unstimulated samples was counted, and the fold of activation calculated (stimulated to unstimulated). Positive cell activation after S protein stimulus was defined as a minimum 2-fold increase over the background (unstimulated) condition.

### 2.6 Statistical methods

Statistical analyses were conducted using R 4.2.2 (19). All alternative hypotheses were two-sided, and statistical tests were performed at a significance level of 0.05. Empirical confidence intervals (CI) of the Wald type, 95%, and two-sided were calculated. All P-values and CI were reported without correction for multiplicity. To test a hypothesis about mean differences, the bootstrap Welch two-sample Student *t*-test on log-transformed data, which considered the variance differences between the two samples, was used. The analysis was conducted utilizing 1000 bootstrap samples (20). For the titre of S-specific antibodies and IgG subclasses for healthy controls and each immunocompromised group, the types of immune response were compared. For the titre of inhibitory antibodies and CD4 T-cells (fold of activation) for each type of immune response, the healthy controls were compared with each immunocompromised group. Spearman’s correlation coefficient (21) was calculated to assess the association between the titres of S-specific IgG and inhibitory antibodies, as well as the titres of S-specific IgG in relation to CD4 T-cells. To test a hypothesis about the correlation coefficient, the one-sample *z*-test with Fisher *z*-transformation was used. This analysis was conducted separately for each type of immune response on log-transformed data for all immunocompromised groups combined.

## 3 Results

### 3.1 Characteristics of study participants

A cohort of 102 immunocompromised children and young adults of the National Institute of Children’s Diseases, Bratislava (Slovakia) and 30 healthy individuals (HC) were included in the analysis (**Table 1**). The IC patients were categorized based on their diagnoses into four groups: PID (**Table E1**); BA-AR (**Table E2**); RD (**Table E3**) and HSCT (**Table E4**). The PID group comprised 17 patients (6 females) with an average age of 14 years. Of them 6 (35%) received the Pfizer mRNA BNT162b2 vaccine, 8 patients (47%) were diagnosed with COVID-19 confirmed by RT-PCR and 3 patients (18%) were vaccinated and overcame SARS-CoV-2 infection (hybrid immunity). The BA-AR group included 36 patients, 15 (42%) were female, with an average age of 15.5 years. In this group, 25 patients (69%) received the Pfizer mRNA vaccine, 9 (25%) were both vaccinated and infected, and 2 patients (6%) were infected. In the RD group, there were 21 children (57% female) with an average age of 12 years. Of these 6 patients (29%) received the Pfizer vaccine, 11 patients (52%) had confirmed SARS-COV-2 infection, and 4 (19%) overcame COVID-19 and were vaccinated. The HSCT group included 28 children aged 5–24 years, of whom 10 (36%) were female. 16 patients (57%) received the Pfizer mRNA BNT162b2 vaccine, 4 patients (14%) were infected and 8 (29%) were both vaccinated and infected. The HC group included 30 individuals (21 females and 9 males) with an average age of 20.2 years. 14 individuals (46%) were vaccinated, 8 (27%) had COVID-19 confirmed by RT-PCR, and 8 (27%) were both vaccinated and infected with the SARS-CoV-2 virus.

**Table 1:** Basic characteristics of study participants.

Diagnosis	Age (years)		Sex		SARS-CoV-2					
	Range	Mean	Male n (%)	Female n (%)	Vaccine n (%)	Infection n (%)	Hybrid* n (%)			
							Total	Inf/vac	Vac/inf	
PID (n=17)	6-24	14.0	11 (65)	6 (35)	6 (35)	8 (47)	3 (18)	2 (12)	1 (6)	
BA-AR (n=36)	6-25	15.5	21 (58)	15 (42)	25 (69)	2 (6)	9 (25)	3 (8)	6 (17)	
RD (n=21)	6-18	12.0	9 (43)	12 (57)	6 (29)	11 (52)	4 (19)	2 (9.5)	2 (9.5)	
HSCT (n=28)	5-24	16.8	18 (64)	10 (36)	16 (57)	4 (14)	8 (29)	6 (22)	2 (7)	
Controls (n=30)	9-26	20.2	9 (30)	21 (70)	14 (46)	8 (27)	8 (27)	4 (13.5)	4 (13.5)	

PID, Primary immunodeficiencies; BA-AR, Bronchial asthma, Allergic rhinitis; RD, Rheumatic disease; HSCT, Hematopoietic stem cell transplantation; *immunity induced by a combination of vaccination and SARS-CoV-2 infection; inf/vac - individuals first infected and then vaccinated; vac/inf - individuals first vaccinated and then infected.

The median time between vaccine administration and blood sampling for the IC group was 90 days (ranging from 8 to 232 days), while the median time between PCR-proven SARS-CoV-2 infection and blood sampling was 59 days (ranging from 14 to 267 days). In the healthy group, the median time from vaccination to blood collection was 76.5 days, with a range of 21 to 191 days. The median time from PCR-confirmed infection to sample collection was 68.5 days, with a range of 15 to 186 days. There was no statistical difference in the intervals between vaccination or infection and sample collection between the IC and HC groups (P = .349, Mann-Whitney test).

All participants in the group of vaccinees received two doses of the vaccine (n=67, 100%). The median time between vaccine doses was 28 days with a range of 21 to 69 days. In the hybrid group (n=32), 28 participants (88%) received two doses, while the remaining four patients (12%) were vaccinated once (two patients with PID, one patient with HSCT, and one patient in the BA-AR group). In the group of infected patients (n=33), only one (3%) from the PID group (XLA) overcame COVID-19 twice; all other infected participants overcame the infection only once (**Tables E1**-**E4**).

In the group of PID patients with induced hybrid immunity (n=3), two individuals were infected before vaccination, while one was vaccinated before infection. Among patients with BA-AR (n=9), three participants were infected and then vaccinated, while six were vaccinated and then infected. In the group of patients with RD (n=4), two were infected prior to vaccination, and the other two were vaccinated prior to infection. In the group of patients who underwent HSCT (n=8), six had infection before vaccination, while two were vaccinated prior to infection. In the control group (n=8), four participants were infected first, and four were vaccinated first (**Table 1**).

Out of 17 PID patients, 11 were undergoing immunoglobulin replacement therapy (IgRT) at the time of sampling, 8 received subcutaneous immunoglobulin (HyQvia) while 3 were treated with intravenous immunoglobulin (Kiovig/Privigen). From the HCST group, only 2 patients were receiving intravenous immunoglobulin (Kiovig/Privigen). Patients who received IgRT were vaccinated at least one week before or after their IgRT treatment (**Table E1**, **E4**).

### 3.2 titres of S-specific antibodies

**Figure 1:**
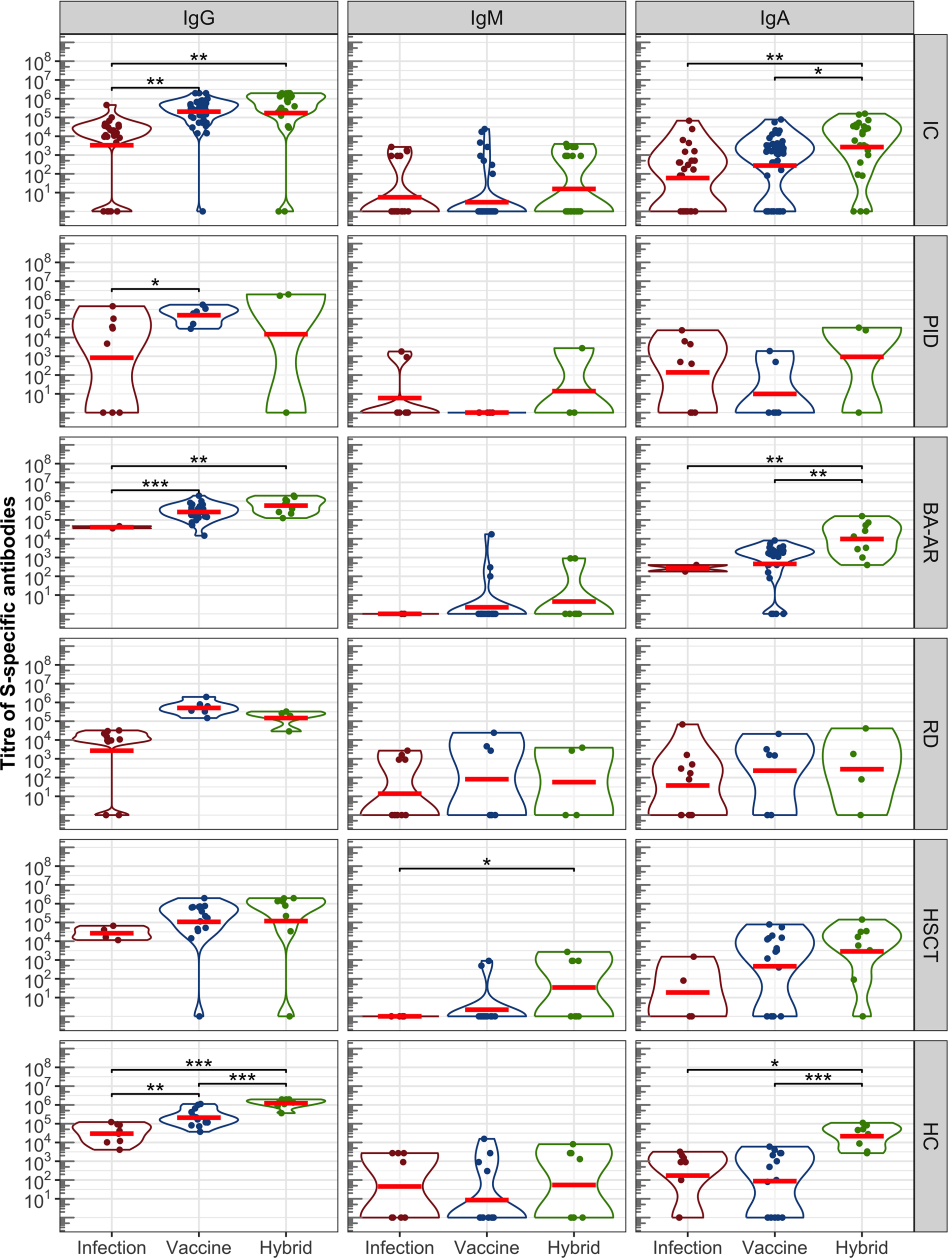
Antibody response induced by SARS-CoV-2 infection, vaccination, and a combination of infection and vaccination (hybrid immune response) evaluated by ELISA. S-specific titres of IgG, IgM, and IgA in a pooled group of IC participants and individual groups that were stratified based on diagnosis —PID,BA-AR,RD, HSCT and HC are shown. The distributions of all variables are visualized using violin plots on a log scale, with the log-transformed data and their geometric mean (red horizontal lines) superimposed on these plots. Statistical significance between individual populations was determined by the bootstrap Welch two-sample Student *t*-test on log-transformed data. For the sake of clarity, only results with statistical significance are shown (*P <.05; **P <.01; ***P <.001). The results are not corrected for multiplicity.

The levels of spike-specific IgG, IgA, and IgM classes of antibodies induced by infection, vaccination, and a combination of both (hybrid antibody response) were determined for the entire immunocompromised group, individual groups stratified based on diagnosis, and a healthy group using ELISA (**Figure 1**).

#### 3.2.1 IgG class

The IgG class reached the highest titres in all groups, regardless of the type of antibody induction (**Figure 1**, **Table E5**). Overall, the IC participants showed significantly higher levels of IgG (expressed as geometric mean titre, GMT) after vaccination compared to those who were infected with SARS-CoV-2 (GMT_vac_ = 205023, 95% confidence interval (CI): 116074–362136 vs GMT_inf_ = 3323, 95% CI: 578-19109, P = .006). The GMT of antibodies induced by hybrid immunity in this group was also notably higher than that observed in the infection cases (GMT_hyb_ = 172819, 95% CI: 33133–901403 vs GMT_inf_ = 3323, 95% CI: 578-19109, P = .001). However, there was no difference between the levels of vaccine- and hybrid immunity-induced IgG levels (P = .849).

The IgG antibody levels in the control HC group followed the expected pattern, the highest IgG response was induced by hybrid immunity (GMT = 1229774, 95% CI: 761791-1985000), followed by the IgG levels after vaccination (GMT = 209327, 95% CI:117764-372082), and the lowest levels were after the infection (GMT = 29623, 95% CI: 10687-82113). The differences were statistically significant (P_hyb/vac_ <.00001, P_vac/inf_ = .002, P_hyb/inf_ <.00001).

Analyses in the individual IC groups confirmed that the IgG titres after vaccination were higher than those after infection (**Figure 1**). These differences were significant only for the PID (GMT_vac_ = 153995, 95% CI:46838-506306, GMT_inf_ = 839, 95% CI:7-100040, P = .038) and BA-AR (GMT_vac_ = 266189, 95% CI:173426-408571, GMT_inf_ = 40915, 95% CI:8049-207983, P <.00001) patients (see also **Table E5**). The low levels of antibodies in the PID group after infection were influenced by the diagnoses of the patients. Of the 8 patients who overcame the infection, only 5 (62.5%) had detectable IgG, while two subjects with XLA and one with cartilage-hair hypoplasia syndrome showed no S-specific IgG levels (**Table E1**). Similarly, higher levels of IgG induced by vaccines were observed in RD and HSCT patients compared to those induced by infection, but without statistical significance (see **Table E5**). Additionally, given the diagnosis and ongoing treatment, two patients (18.2%) from the RD group (**Table E3**, patients no. 3 and 10) who overcame the infection as well as one vaccinated patient after HSCT (6.3%, **Table E4**, patient no. 19) were unable to develop S-specific IgG.

The combined stimulation of immune system through vaccination and infection (hybrid immunity) resulted in the generation of IgG in most IC subjects, except for one case in the PID group (33.3%) and another in the HSCT (12.5%) group (**Figure 1**, IgG). Both individuals received two doses of the Pfizer mRNA vaccine and tested positive for SARS-CoV-2 by RT-PCR. This lack of antibody response can be attributed to the diagnosis of the PID patient (nude severe combined immunodeficiency) and the ongoing therapeutic regimen for the HSCT patient, which involves corticosteroid administration and anti-TNF therapy with adalimumab.

Overall, a combination of vaccination and infection did not lead to a statistically significant increase in anti-S antibody production in comparison to vaccination in all groups of IC participants. A trend was observed in the BA-AR group (GMT_hyb_ = 584314, 95% CI:276387–1235000 and GMT_vac_ = 266189, 95% CI:173426-408571), which was not statistically significant (P = .066).

The levels of hybrid-induced antibodies were higher than infection-induced antibody levels in the IC groups, but only in the BA-AR group this difference was statistically significant (GMT_inf_ = 40915, 95% CI: 8049-207983, GMT_hyb_ = 584314, 95% CI:276387-1235000, P = .002) (see **Table E5**). The difference in the PID, RD, and HSCT groups did not reach statistical significance (**Figure 1**, see **Table E5**).

#### 3.2.2 IgA class

Overall, the titre of IgA reached lower levels compared to IgG in all groups (**Figure 1**). In the combined IC group, 72.5% patients showed detectable IgA response, and 76.7% in the HC group. In the IC group, the highest titres of IgA were detected after combined (hybrid) activation of the immune system (GMT_hyb_ = 2672, 95% CI: 566-12623), followed by the levels of vaccine-induced IgA (GMP_vac_ = 275, 95% CI: 97-777), and lowest levels were observed after infection (GMT_inf_ = 60, 95% CI: 13-280). The differences were significant between the hybrid and vaccine induced levels (P = .016) and between the hybrid and infection induced levels (P = .002). This pattern of IgA levels was similar to that observed in the HC group, where the hybrid immunity led to the highest IgA response (GMT_hyb_ = 21548, 95% CI:6472-71744) significantly different from those in vaccinated (GMT_vacc_ = 87, 95% CI:10-726, P <.00001) and infected (GMT_inf_ = 172, 95% CI: 11-2809, P = .020) participants. The analysis in the individual groups of the immunocompromised participants showed that these differences in the IgA levels were driven by the BA-AR group. The highest levels were observed after combination of vaccination and infection (GMT_hyb_ = 9726, 95% CI: 1999-47310) and were significantly different from those induced either by vaccination (GMT_vac_ = 456, 95% CI:137-1516, P = .007) or infection (GMT_inf_ = 268, 95% CI:2-42839, P = .003) alone. No difference was observed between vaccination and infection induced IgA levels (P = .44). The IC subgroups did not show significant differences between IgA levels (**Table E5**). All patients in the BA-AR group generated hybrid IgA like the healthy control, while one patient from the PID, RD, and the HSCT groups was a non-responder (**Figure 1**).

#### 3.2.3 IgM class

The overall IgM antibody response in the entire IC group was very low (22.5% for IC, 56.7% for HC) compared to that of IgG and IgA across all analyzed groups, which is also influenced by the time of sample collection (Mean_HC_ was 73.7 days, 95% CI:53.04-94.4; Mean_IC_ was 88 days, 95% CI:75.7-100.3, P = .35). Additionally, a large portion of participants did not exhibit appreciable levels of antibodies, regardless of the type of immunity. The GMT of IgM in the immunocompromised group was 3 (95% CI: 1-7) for the vaccine, 6 (95% CI: 2-21) for infection and 16 (95% CI: 3-73) for hybrid; slightly higher GMTs were observed in the control group (see **Table E5**).

### 3.3 S-specific IgG subclass determination

**Figure 2:**
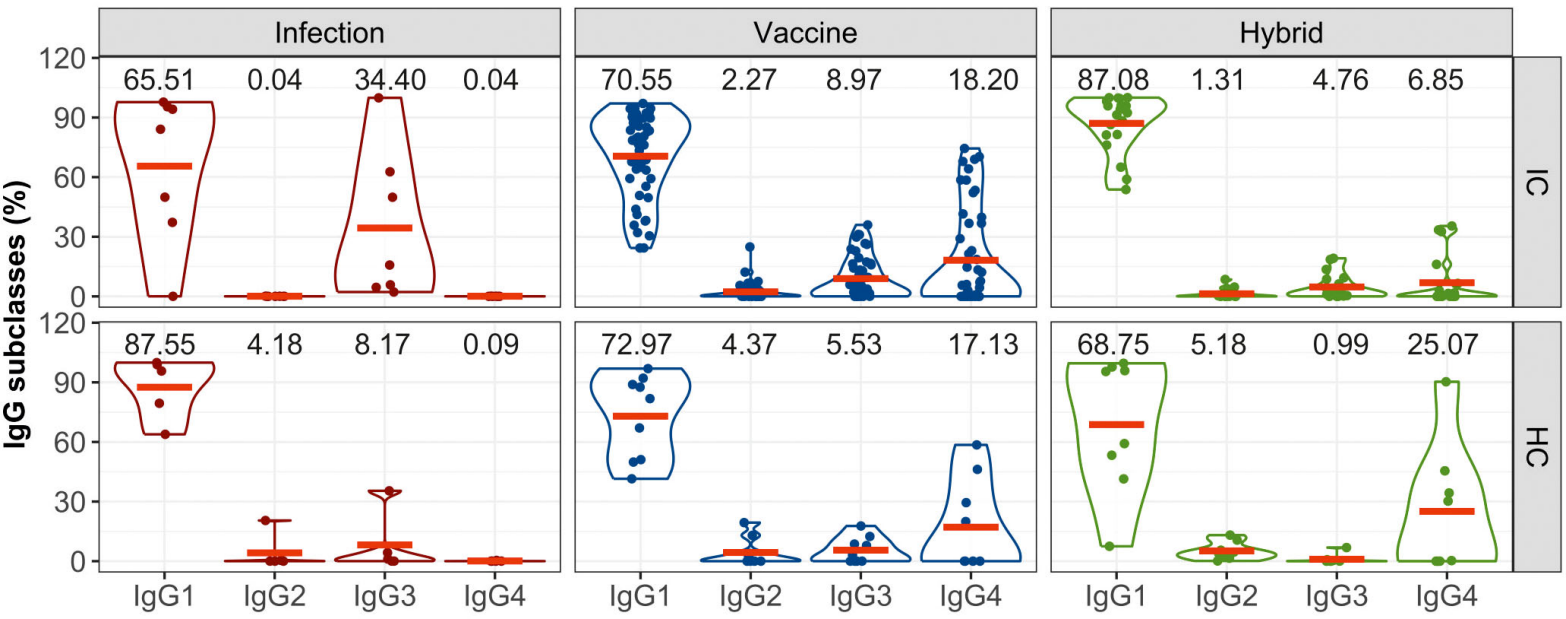
The proportion of S-specific IgG subclasses within the total IgG response for the IC and HC groups after infection-, vaccine- and hybrid-induced immunity. IgG subclasses (IgG1, IgG2, IgG3, IgG4) are expressed as percentages of total IgG. The distributions of all variables are visualized using violin plots and their means (red lines) are superimposed on these plots. For the sake of clarity, the means are also expressed as numerical values.

The analysis of the subclasses of S-specific IgG revealed a predominant induction of IgG1 in both IC and HC groups irrespective of the type of the inducing immunity (**Figure 2**). In the IC group, infection also led to the generation of IgG3 in some patients; in the healthy group, IgG3 and IgG2 were induced in one participant each. In the immunocompromised group, IgG3 constituted up to 34% of the total IgG which is higher than 8% observed in the healthy group. Conversely, vaccination and hybrid stimulation of the immune response resulted in the production of all IgG subclasses. The analysis revealed a similar profile of IgG subclasses induced by the vaccine in both immunocompromised and healthy groups, with a relatively notable proportion of IgG4 antibodies (18% in the immunocompromised group and 17% in the healthy group). After the hybrid stimulation of the antibody response, the amount of IgG4 increased in healthy controls reaching 25%, while in the immunocompromised group, it remained at 7%.

### 3.4 titres of inhibitory antibodies

**Figure 3:**
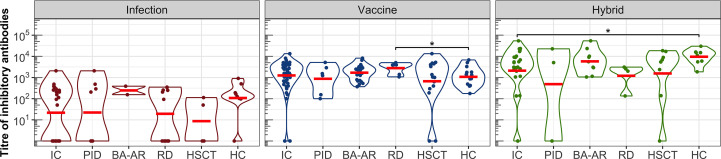
The titres of inhibitory antibodies in the entire IC group, in the individual diagnosis-stratified groups (PID, BA-AR, RD, and HSCT), and in the healthy participants are shown using violin plots on a logarithmic scale. Geometric means are indicated by red horizontal lines. Statistical significance between individual groups was assessed using the bootstrap Welch two-sample Student’s *t*-test on log-transformed data. Differences that reach statistical significance between the HC and IC groups are marked with an asterisk (*P <.05).

Next, we analyzed the ability of plasma samples to block the interaction between the S protein of SARS-CoV-2 and ACE2 expressed on the surface of permissive cells (**Figure 3**, **Table E6**). All individuals who developed vaccine-induced or hybrid-induced IgG antibodies showed detectable levels of inhibitory antibodies (except one vaccinated patient in the HSCT group despite the IgG titre of 45500, **Figures 1**, **3**).

After infection we found relatively low titres of inhibitory antibodies in immunocompromised and healthy individuals. Several participants generated detectable titres of S-specific IgG, yet had undetectable levels of inhibitory antibodies: one individual in the HC group (IgG titre of 12000), one in the PID group (IgG titre of 4700), three individuals in the RD group (IgG titres of 32400, 9500 and 8300), and two in the HSCT group (**Figure 3**). The GMT of the inhibitory antibodies for the entire IC group was only 22 (95% CI: 7-71), while for the HC group it was 108 (95% CI: 19-597). These values were lower compared to those of the vaccine and hybrid immunity-induced inhibitory antibodies (**Figure 3**, **Table E6**). After vaccination, GMT for IC patients was 1252 (95% CI: 770-2036), which was similar to the GMT of HC individuals (GMT = 1077, 95% CI: 586-1981).

**Figure 4:**
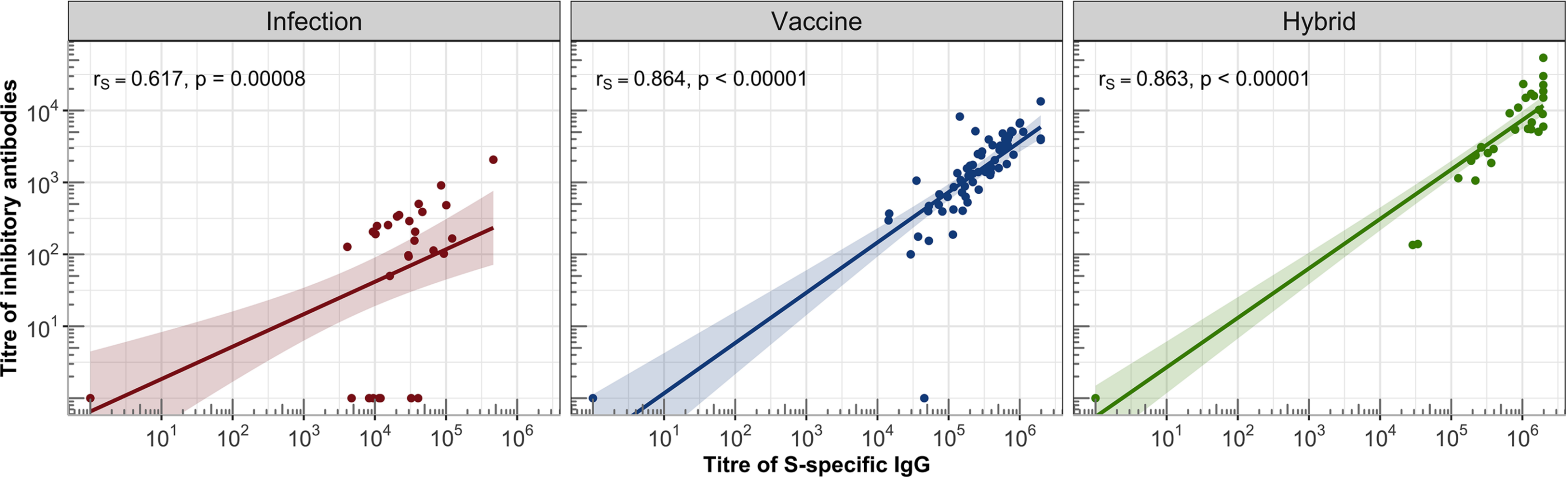
Correlations between the titres of IgG and inhibitory antibodies were assessed in the IC and HC groups. For each group, a linear regression line with 95% confidence band was plotted on a logarithmic scale. Spearman’s correlation coefficient (r_s_) was calculated to evaluate the association between S-specific IgG titres and inhibitory antibody titres, along with the corresponding statistical significance based on the P-value.

The hybrid immunity induced significantly higher GMT in the healthy individuals (GMT = 9601, 95%CI: 4668-19747) than in the total IC group (GMT = 2108, 95% CI: 658-6760, P = .041), which was caused by the presence of two non-responding patients in the IC group (they also had IgG titres below detection levels). The analysis of the individual IC subgroups showed that the BA-AR patients reached the highest and most consistent levels of inhibitory antibodies compared to the PID, RD, and HSCT subgroups induced by the infection, vaccine or hybrid immunity (**Figure 3**, **Table E6**). The S-specific IgG levels induced by vaccination and hybrid immunity positively correlated with the titres of inhibitory antibodies blocking the S-ACE2 interaction (vaccine IgG: r = 0.864, P <.00001; hybrid immunity IgG: r = 0.863, P <.00001) (**Figure 4**). We also observed a significant positive correlation between the infection-induced S-specific IgG levels and inhibitory antibodies (r = 0.617; P = .00008), however, the relationship was weaker than for vaccine- and hybrid immunity-induced antibodies.

### 3.5 S-specific CD4 T-cells response

**Figure 5:**
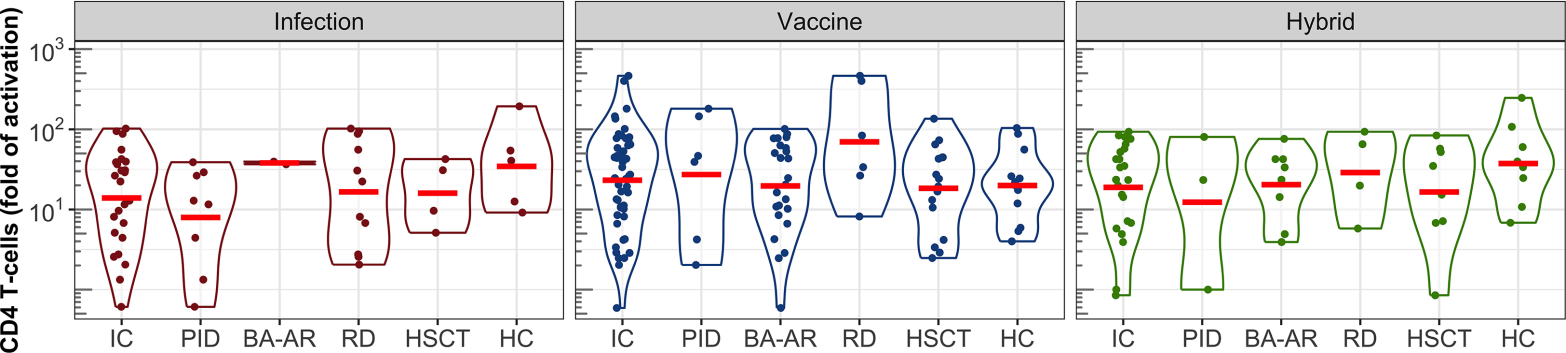
S-specific CD4 T-cell responses following infection, vaccination, and hybrid exposure. Fold changes in T-cell activation are shown in violin plots on a logarithmic scale. Data are presented for the pooled IC participants, individual subgroups (PID, BA-AR, RD, and HSCT) and the HC group. Geometric means are indicated by red horizontal lines.

Helper T-cells play an essential role in the development and affinity maturation of antibodies. We analyzed the levels of CD4 T-cells induced by vaccination, infection, and their combination in immunocompromised and healthy individuals using the FASCIA method in fresh whole blood (17, 18, 23, 24). Stimulation of the blood cells (fold of activation) with the S protein led to the induction of the S-specific CD4 T-cells in all healthy individuals (**Figure 5**).

Comparable levels of CD4 T-cell activation were observed across individual groups, including healthy controls, with no statistically significant differences (**Figure 5**). Additionally, the type of immune response induction, whether due to infection, vaccination, or a combination of vaccination and infection, did not significantly affect the levels of the S protein-specific CD4 T-cells, as demonstrated by the geometric mean values (GMV, **Table E7**). For healthy individuals who were infected, the GMV was 35 (95% CI: 8-158), while for the IC patients who were infected, it was 14 (95% CI: 8-25). The GMV of the CD4 T-cell response to the vaccine in healthy controls was 20 (95% CI: 10-39), and was similar to that in the combined group of the IC patients (GMV = 23, 95% CI: 16-35). The IC group contained two patients with the strongest T-cell response among all participants (both belonged to the RD group) with activation levels of 402-fold and 465-fold. The hybrid CD4 T-cell response reached the GMV of 37 (95% CI: 14-99) in the healthy group, and 19 (95% CI: 11-34) in the IC group.

Additionally, we found a weak correlation between vaccine-induced S-specific CD4 T-cell responses and S-specific IgG responses when considering all individuals combined (r = 0.333; P = .0057). The correlations in the infected and hybrid groups were not statistically significant (see **Figure E1**). This limited correlation highlights a mismatch between humoral and cellular responses in some patients with immune deficiencies.

### 3.6 Profile of immune responses across immunodeficiencies

**Figure 6:**
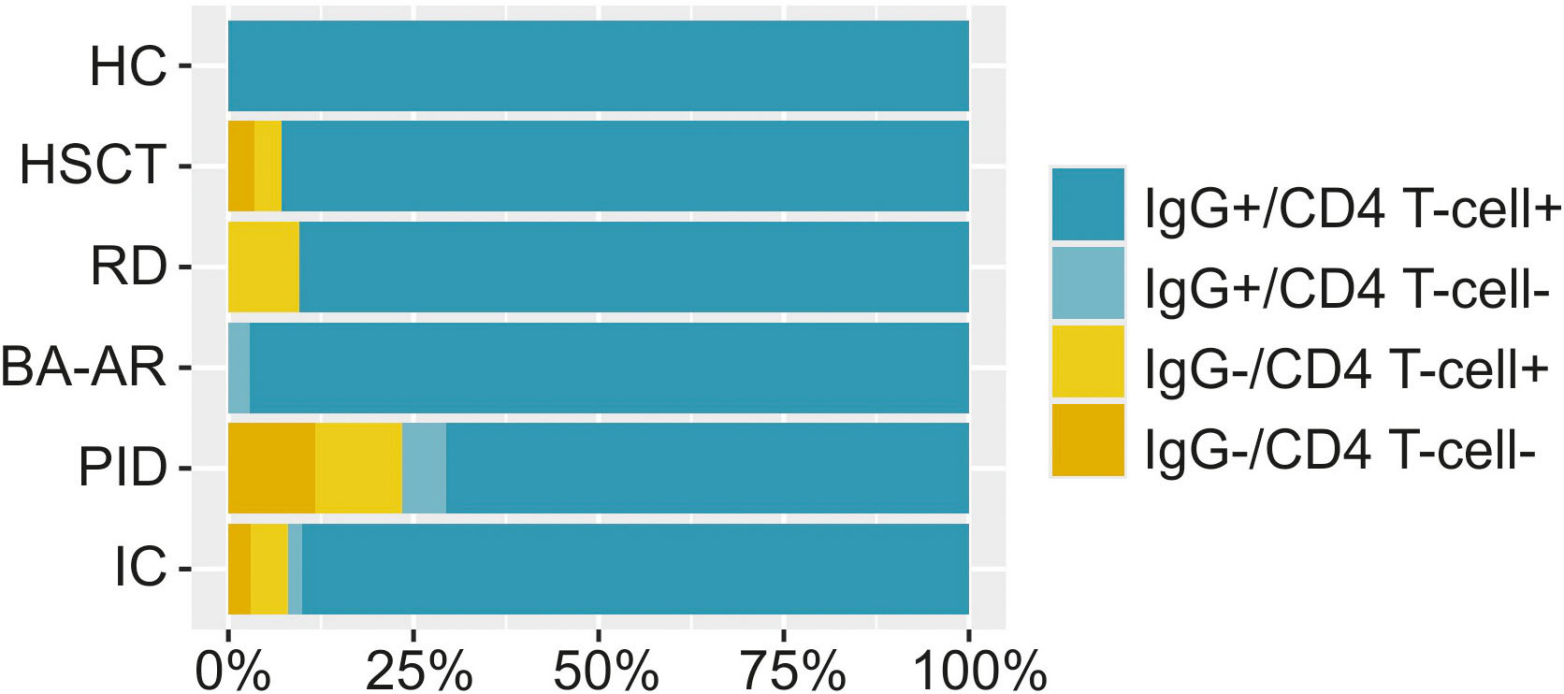
The percentage of individuals with S-specific IgG and/or S-specific CD4 T-cells is displayed. The relative frequencies are illustrated using bar plots scaled to 100% for the entire immunocompromised group (IC, n = 102), individual diagnostic groups (n = 28, responders 93%; RD, n = 21, responders 90%; BA-AR, n = 36, responders 97%; PID, n = 17, responders 71%) and the HC group (n = 30, responders 100%). A responder was defined as an individual capable of reacting to immune stimuli by generating at least one type of immune response, either humoral or cellular.

Using the collected data on antibody and cell responses, we assessed the proportions of responders and non-responders (S-specific IgG titres and fold activation of CD4 T-cells) in all groups after vaccination, infection, and hybrid-induced immunity (**Figure 6**). In this context, a responder was defined as an individual who can react to stimuli – such as a vaccine, an infection, or a combination of both – by generating at least one type of immune response.

All healthy individuals generated both antibody and cell responses, regardless of the nature of the inducer of the immune response. Unsurprisingly, the proportion of non-responders that failed to mount either humoral or cellular response was highest in the PID group (29%). Among these, two were diagnosed with XLA and after infection did not mount detectable IgG response but exhibited CD4 T-cell responses (39-fold and 12-fold activation). The other two patients had cartilage-hair hypoplasia syndrome and FOXN1 deficiency and they were unable to generate any IgG or cellular response after infection or a combination of vaccination and infection. Additionally, one patient with combined immunodeficiency had relatively low levels of antibodies (IgG titre of 4700) and did not develop detectable CD4 T-cells response. In the BA-AR group, only one patient did not mount detectable CD4 T-cells response (3%). In the RD group, we found two individuals with negative humoral responses (10%), one also with a borderline-positive cellular immune response. Furthermore, in the HSCT group, one patient (with hybrid immunity) showed undetectable levels of both antibodies and CD4 T-cell activation, while another patient failed to show an antibody response (7%).

### 3.7 Immune response of patients with IgRT therapy

Out of 17 PID patients, 11 were receiving IgRT during the duration of the study. Among them, five were from the infected group, four from the vaccinated group, and two from the hybrid group. In two patients with XLA, we did not detect any specific antibodies following viral infection; however, S-specific CD4 T-cells were significantly stimulated in these patients (fold of activation 39 and 12, respectively). In a patient with CID, we observed relatively low titres of S-specific IgG, IgM, and IgA antibodies (titre of 4700, 900, and 400, respectively) and no activation of CD4 T-cells. Additionally, a patient diagnosed with Cartilage-Hair Hypoplasia syndrome did not exhibit any immune response, and despite receiving IgRT (Kiovig/Privigen), no antibodies were detected. However, this patient passed away due to complications associated with COVID-19, specifically respiratory failure. In contrast, another patient showed relatively high levels of IgG and IgA (titre of 100800 and 4400, respectively) and CD4 T-cells (fold of activation 4).

In four IgRT-receiving patients in the vaccinated group, we observed IgG titres ranging from 29300 to 557500 and activated CD4 T-cells (fold of activation from 4 to 181).

There were two patients in the hybrid group receiving IgRT therapy. In one of these patients, no S-specific antibodies or T-cells were detected, even after being vaccinated twice and subsequently infected. Conversely, the second patient displayed very high levels of antibodies (IgG titre of 1684300; IgA titre of 33400) and significant cellular immunity (fold of activation 80).

During the sampling period, only two patients from the HSCT group who received the vaccine were undergoing IgRT. In one patient, the IgG titre of 51700 and significant activation of CD4 T-cells (fold of activation 136) were observed. In the second patient, the vaccination did not induce an antibody response, only a cellular response, as indicated by CD4 T-cell activation with a fold of activation of 24.

## 4 Discussion

This study aimed to evaluate how various primary and secondary immunodeficiencies influence the immune response to SARS-CoV-2 infection, mRNA vaccination, or a combination of both. The cohort included 102 immunocompromised children, adolescents and young adults with conditions such as primary immunodeficiencies, asthma/allergic rhinitis, rheumatoid diseases, and those who had hematopoietic stem cell transplants. The study assessed antibody responses and CD4 T-cell responses, focusing on the titres of antibody isotypes targeting the S protein (IgG, IgM, IgA, and IgG subclasses IgG1, IgG2, IgG3, and IgG4). It also evaluated the neutralization capacity of these antibodies and their correlation with S-specific binding antibody titres.

In our study, we demonstrated that majority of immunocompromised patients developed a diverse range of matured, multi-isotype antibody responses specific to the S protein, induced by vaccination, infection, or a combination of both. Among these inducers, infection resulted in the lowest levels of S-specific antibodies. This finding aligns with a previous study by Lafon et al. (25), which reported that convalescent COVID-19 patients had lower levels of antibodies compared to those receiving the mRNA-1273, BNT162b2, or ChAdOx1 vaccines. Similarly, de Gier et al. (26) found that the concentration of S-specific antibodies was lower in infection-induced immunity compared to vaccine-induced or hybrid immunity. Vaccination combined with SARS-CoV-2 infection creates hybrid immunity, resulting in the highest levels of S-specific antibodies. We observed an IgG response to the S protein in most IC patients, which indicates that immune mechanisms are functioning, leading to secondary high-affinity IgG responses. Since we did not identify any individuals with only primary IgM responses, we can assume that class switching to high-affinity secondary IgGs was not impaired by immunodeficiencies (except where disease directly thwarts B-cell responses). This finding is extremely important from the perspective of antiviral immunity in immunocompromised patients.

Our previous research demonstrated that S-specific antibodies capable of inhibiting the S-ACE2 interaction exhibited strong neutralizing activities against the SARS-CoV-2 virus (16). Consequently, the S-ACE2 inhibition assay indicated that vaccination, viral infection, and the combination of vaccination and infection all induced high-quality antibodies with neutralizing activity. Importantly, our data revealed a significant positive relationship between S-specific IgG levels and the corresponding neutralization activities for all three types of stimuli (infection, vaccination, and vaccination plus infection). These results suggest that the induced antibody response has a protective nature and is a crucial component of antiviral immunity. We suppose that even antibodies binding to the S protein but not neutralizing SARS-CoV-2 may still play a beneficial role in immune control of the infection. It is important to note that, beyond neutralization, high-affinity IgG antibodies are associated with a wide range of Fc-dependent effector functions, making them a crucial component of protection against SARS-CoV-2 infection (27–29). In this context, IgG1 and IgG3 are powerful pro-inflammatory antibodies capable of inducing effector functions, whereas IgG2 and IgG4 are considered anti-inflammatory with a limited capacity to mediate such functions (30, 31).

During the sampling period of the ongoing study (July 2021 to May 2022), IgRT products were shown to contain varying concentrations of SARS-CoV-2 antibodies (32, 33). We did not have the possibility to determine the levels of the anti-S antibodies in the IgRT preparations, therefore, it was not possible to accurately assess the proportion of IgRT-derived antibodies in the plasma of the study participants. In some cases, despite administering IgRT, we found no S-specific antibodies, and in PID patients (XLA) we only detected the S-specific CD4 T-cells. In other instances, we observed specific IgG antibodies, as well as IgA and CD4 T-cells. The levels of IgA antibodies in IgRT are relatively low with short half-lives (34), which contrasts with the significantly high IgA titre seen in a patient with CVID following an infection and subsequent vaccination. This indicates that IgA and CD4 T-cells were generated following immune system stimulation due to infection or vaccination. Based on these observations, we assume that the antibodies observed in patients undergoing IgRT therapy primarily result from immune response to infection or vaccination, and the contribution of IgG antibodies from IgRT is minimal. Similarly, recent studies have indicated that none of the IgRT products significantly impact overall antibody levels (12, 33, 35).

Given the important role of IgG antibodies in antiviral immunity, we analyzed the abundance of anti-S IgG subclasses. As expected, the analysis revealed the predominant presence of pro-inflammatory S-specific IgG1 across all groups. We observed unexpectedly high levels of IgG3 in IC patients following infection. In three participants the proportion of IgG3 ranged from 50% to 98%, even though the total levels of IgG antibodies were relatively low. Since the class switching is regulated by cytokines, we hypothesize that changes in the balance of the cytokine environment due to anti-inflammatory therapy may affect the class switching to the IgG3 subclass. We also detected the S-specific IgG4 subclass in individuals with both vaccine-induced and hybrid-induced response. Interestingly, no S-specific IgG4 antibodies were found in individuals with only infection- induced immunity. In vaccinated IC and HC individuals, we observed a switch in the antibody response to IgG4 in those tested more than 58 days after receiving two vaccinations (ranging from 58 to 232 days, with an average of 140 days). These findings are in line with a recent study indicating the emergence of S-specific IgG4 antibodies in the sera of individuals 5 to 7 months after their second vaccine dose (36). In the hybrid immunity group (IC and HC), after two vaccine doses, SARS-CoV-2 infection probably acted like a third dose, promoting the induction of IgG4. Boosting of vaccine-induced immune memory by SARS-CoV-2 infection was already reported to induce a switch in the antibody response to IgG4 (36). However, the implications of this class switching from pro-inflammatory IgG1 to anti-inflammatory IgG4 for antiviral defense remain unclear.

To provide a comprehensive overview of induced antibodies, we measured the levels of IgM and IgA in subjects with vaccine-induced, infection-induced, and hybrid immunity. Consistent with findings from other studies, we observed that more than half of the participants had very low or undetectable IgM levels (37–39), which may reflect the kinetics of IgM in plasma following antigenic stimulation. Thus, one possible explanation for the low IgM levels could be an extended interval between IgM induction and blood collection.

We found that IgA levels did not reach the levels observed for IgG, despite the significant role that IgA antibodies play in viral immunity. We observed relatively low IgA levels induced by SARS-CoV-2 infection; however, vaccination followed by infection resulted in a notable increase in IgA levels, which could be beneficial for the patient.

All participants in the group of vaccinees received two doses of the vaccine, although it may not be sufficient to induce an adequate immune response, particularly for immunocompromised patients. Several studies indicated that the effectiveness of immune response following the third and fourth doses of the vaccine varies based on the degree of immune impairment of the patient (33, 35, 40) and a recent publication demonstrated that booster vaccinations significantly enhanced the immune response in patients with inherited immune deficiencies with a milder clinical phenotype (12). Therefore, it will be important to identify which patients benefit most from additional vaccine doses. Our data showed that, regardless of diagnosis, the majority of patients developed antibody and helper T-cell responses comparable to those of healthy controls. Only patients with serious immune system defects or those undergoing immunosuppressive therapy were unable to respond adequately to the immune stimulators. Four patients with secondary immunodeficiencies, two with RD and two who had HSCT, did not mount an antibody response. The patients with RD included one with juvenile dermatomyositis and one with juvenile idiopathic arthritis and both were treated with methotrexate at the time of sampling. Of the two HSCT patients, one was diagnosed with B-cell acute lymphoblastic leukemia (B-ALL) and was receiving CAR T-cell immunotherapy, and the other had an X-linked inhibitor of apoptosis protein deficiency and was treated with cyclosporine A and hydrocortisone. These therapies likely inhibited the antibody production. These findings are in line with previously published studies, which suggest that immune dysregulation in patients leads to suboptimal adaptive immunity (8–11, 41, 42). The individuals with undetectable antibodies or CD4 T-cells were found only in the immunocompromised groups, not between healthy participants, which is consistent with earlier reports (41, 43).

As expected, we did not detect any antibody response in SARS-CoV-2-infected patients with XLA; however, this antibody deficiency was compensated by an S-specific CD4 T-cell response. Several other studies have documented induced S-specific T-cells in children with XLA (11, 44–46). CD4 helper T-cells are multipotent and play a critical role in protecting against SARS-CoV-2 infection by performing a wide range of helper and effector functions (47). They promote the proliferation and differentiation of CD8 T-cells into effector cytotoxic cells, and they also have direct antiviral activities or can differentiate into effector cells, such as Th1-cells (48, 49). Since patients with XLA were able to overcome SARS-CoV-2 infection, it seems likely that the virus-induced CD4 T-cells provided substantial antiviral immunity. The significant role of T-cells in immunity is further supported by case reports from Soresina et al. (50), which described two patients with XLA who overcame severe COVID-19 without mounting an antibody response. These findings highlight the important role of T-cells in virus protection and suggest that a lack of antibody response does not necessarily indicate a lack of immunity.

This is a cross-sectional study with several limitations. The nature of the study did not allow us to assess the temporal dynamics of the immune response. Our cohort is highly heterogeneous, comprising a broad range of diagnoses and ongoing therapies, which may affect the outcomes to varying degrees. 11 patients with PID, as well as two with HSCT, have been on IgRT therapy. This treatment may have affected the levels of antibodies detected in some cases. Additionally, the individual IC groups included vaccinated individuals, infected individuals, and vaccinated individuals who have recovered from a SARS-CoV-2 infection. Although all participants in the vaccine group received two doses of the vaccine, in the hybrid group 12% received only one dose.

The small number of patients in each IC group makes it impossible to draw definitive conclusions. Variations in the time intervals between the immune stimulation and blood collection was not considered in the statistical analysis and might have contributed to high variability in the measured components of the immune response. The study did not assess the CD8 T-cell response, which is crucial due to its direct antiviral cytotoxic activity and is necessary to obtain a comprehensive understanding of the induced immunity.

In conclusion, our data suggest that antibody-mediated immunity is generated in a diverse group of immunocompromised patients after vaccination and infection, indicating their potential to provide effective protection against SARS-CoV-2, and possibly against other infectious pathogens. Furthermore, patients with impaired antibody production due to immunodeficiency exhibited activated T-cell-mediated immunity, which likely played a role in overcoming SARS-CoV-2 infection.

## Data Availability

The original contributions presented in the study are included in the article/**Supplementary Material**. Further inquiries can be directed to the corresponding author.
