# Supplementary material for: Impact of immunodeficiencies on immunity induced by SARS-CoV-2 infection, mRNA BNT162b2 vaccination, and their combination in children and young adults
Source: Front Immunol. 2025 Nov 7;16:1661282. doi: 10.3389/fimmu.2025.1661282 (PMC12634547; doi:10.3389/fimmu.2025.1661282)
Supplement: Supplementary file 1 [file DataSheet1.docx]

Table E1: Characterization of patients with primary immunodeficiencies included in the study

| Patient no. | Age (Year) | Sex | Diagnosis | Clinical phenotype/ comorbidities | Auto  immunity (Y/N) | Genetics | IgRT/ type | IgG (IU/ml) | CD3 (cells/µL) | SARS-CoV-2 vaccine | SARS-CoV-2 infection | days |
| --- | --- | --- | --- | --- | --- | --- | --- | --- | --- | --- | --- | --- |
| 1 | 17 | M | XLA | RRTI, bronchiectasis | N | ND | SCIG | 6.81 | 1.9 | 0 | 1 | 204 |
| 2 | 13 | M | XLA | RRTI, furunculosis of face | N | mutation in Btk gene c.604_607delACAG | SCIG | 8.75 | 1.1 | 0 | 2 | 60 |
| 3 | 13 | M | NFkB1 haploinsufficiency | bicytopenia - ITP, AIN, gingivitis | Y | NFkB1 haploinsufficiency (C>T3ntEx10) | 0 | 8.5 | 0.44 | 0 | 1 | 58 |
| 4 | 15 | M | CID | RRTI | N | ND | SCIG | 10.44 | 0.15 | 0 | 1 | 69 |
| 5 | 6 | F | Kabuki syndrome 1 | hearing impairtment, speech impairment, strabism, hypermetropy | N | de novo variant in gene KMT2D c.13885A>C (p.Thr4629Pro) | 0 | 7.64 | 1.98 | 0 | 1 | 87 |
| 6 | 6 | M | DiGeorge syndrome | facial dysmorphia, cardiac defect, speech impairment | N | del 22q11.2 | 0 | 9.87 | 0.43 | 0 | 1 | 134 |
| 7 | 15 | F | Cartilage hair hypoplasia syndrome | infections, chondrodysplasia, celiac disease, AIHA | Y | ND | IVIG | 4.72 | 0.79 | 0 | 1 | 267 |
| 8 | 14 | M | undetermined - laboratory IgG deficiency, MPO deficiency | RRTI, IUGR, short stature, skeletal dysplasia, eczema | N | ND | IVIG | 9.76 | 1.2 | 0 | 1 | 142 |
| 9 | 15 | M | CVID | RRTI | N | ND | 0 | 4.13 | 1.36 | 2 | 0 | 126 |
| 10 | 24 | F | CVID | RRTI, vitiligo, reccurent cystitis and vulvovaginitis | N | ND | SCIG | 7.62 | 3.56 | 2 | 0 | 21 |
| 11 | 19 | M | CVID | RRTI, otitis, IBD-like | Y | ND | SCIG | 7.16 | 1.6 | 2 | 0 | 13 |
| 12 | 17 | F | CVID | RRTI | Y | ND | SCIG | 7.14 | 1.4 | 2 | 0 | 194 |
| 13 | 21 | F | APDS | RRTI, otitis, vulvitis, bone dysplasia | N | ND | IVIG | 8.86 | 0.61 | 2 | 0 | 98 |
| 14 | 12 | M | Hypereosinophilic syndrome due to somatic mutations in STAT5b | atopic dermatitis, recidivic viral infection, polyvalent sensibilisation | N | STAT5B, GOF heterozygot, c.2092_2095delGGAT, p.Gly698Thrfs*2, ex17 | 0 | 12.85 | 1.06 | 2 | 0 | 58 |
| 15 | 11 | M | CVID | RRTI | N | ND | SCIG | 8.44 | 1.1 | 1 | 1 | 21 |
| 16 | 14 | M | Nude SCID - FOXN1 deficiency | infections, Burkitt leukemia, hypersplenism | N | compound heterozygote - missense p.P350L (c.1049C>T NM_003593.2) + p.C82* (c.246C>A NM_003593.2) | SCIG | 8.72 | 0.5 | 2 | 1 | 29 |
| 17 | 14 | F | FCAS2 | spleen abscesses, erythema nodosum/rash, hepatoslenomegaly, reccurent fevers | N | missense mutation in gene NLRP12, Arg352Cys | 0 | 11.16 | 2.4 | 1 | 1 | 21 |

M-male; F-female; XLA-X-linked agammaglobulinemia; CID-Combined immunodeficiency; CVID-Common variable immunodeficiency; APDS-Activated PI3K delta syndrome; FCAS2-Familial cold autoinflammatory syndrome 2; SCID-Severe combined immunodeficiency; RRTI-Recurrent respiratory tract infections; IUGR -Intrauterine growth restriction; ITP- Immune thrombocytopenia; IBD-like - Inflammatory bowel disease like; AIN-Autoimmune neutropenia; AIHA-Autoimmune hemolytic anemia; SCIG -Subcutaneous immunoglobulin (HyQvia) ; IVIG - Intravenous immunoglobulin (Kiovig/Privigen); Y-yes, N-no; ND - not determined; NT - not tested; IgRT - Immunoglobulin replacement therapy; IgG - Immunoglobulin class G; 0 - no therapy, or without infection; 1 - one dose of vaccine, or infection; 2 - two doses of vaccine; days-time from vaccination to blood collection or time from PCR-confirmed infection to blood collection

Table E2: Characterization of patients with bronchial asthma and allergic rhinitis included in the study

| Patient no. | Age (Years) | Sex | Diagnosis | IgE (normal range 0-200 IU/ml) | SARS-CoV-2 vaccine | SARS-CoV-2 infection | days |
| --- | --- | --- | --- | --- | --- | --- | --- |
| 1 | 14 | M | BA | 305.2 | 0 | 1 | 197 |
| 2 | 6 | M | BA, AR | 63.1 | 0 | 1 | 44 |
| 3 | 16 | M | BA, AR | 7 | 2 | 0 | 74 |
| 4 | 13 | M | AR | 675.3 | 2 | 0 | 46 |
| 5 | 17 | M | BA, AR | 11.7 | 2 | 0 | 155 |
| 6 | 17 | M | BA, AR | 39.3 | 2 | 0 | 155 |
| 7 | 18 | M | BA, AR | 44.1 | 2 | 0 | 81 |
| 8 | 14 | F | BA, AR | 52.7 | 2 | 0 | 70 |
| 9 | 18 | M | BA | 66.7 | 2 | 0 | 131 |
| 10 | 12 | M | AR | 213.2 | 2 | 0 | 19 |
| 11 | 12 | M | BA, AR | 162.2 | 2 | 0 | 58 |
| 12 | 15 | F | AR | 59.5 | 2 | 0 | 87 |
| 13 | 12 | F | BA, AR | 176.8 | 2 | 0 | 8 |
| 14 | 18 | F | BA | 86.1 | 2 | 0 | 151 |
| 15 | 15 | F | BA | 14.8 | 2 | 0 | 157 |
| 16 | 12 | F | BA | 53.7 | 2 | 0 | 98 |
| 17 | 18 | F | BA | NT | 2 | 0 | 157 |
| 18 | 25 | M | BA | NT | 2 | 0 | 206 |
| 19 | 16 | M | BA, AR | 342.7 | 2 | 0 | 119 |
| 20 | 14 | F | BA, AR | 227.5 | 2 | 0 | 123 |
| 21 | 16 | M | BA, AR | 849 | 2 | 0 | 142 |
| 22 | 13 | M | BA | 117.1 | 2 | 0 | 126 |
| 23 | 18 | M | AR | NT | 2 | 0 | 81 |
| 24 | 14 | F | BA, AR | 12.8 | 2 | 0 | 19 |
| 25 | 16 | M | BA, AR | 1574.8 | 2 | 0 | 90 |
| 26 | 17 | M | BA, AR | 107.3 | 2 | 0 | 86 |
| 27 | 14 | M | BA, AR | 299.7 | 2 | 0 | 94 |
| 28 | 13 | F | BA, AR | 16.8 | 2 | 1 | 18 |
| 29 | 18 | F | BA | <7,0 | 2 | 1 | 132 |
| 30 | 14 | F | AR | 10.1 | 1 | 1 | 83 |
| 31 | 17 | F | BA | >2000.0 | 2 | 1 | 100 |
| 32 | 23 | F | AR | NT | 2 | 1 | 220 |
| 33 | 16 | M | BA, AR | 1672.1 | 2 | 1 | 13 |
| 34 | 16 | M | AR | 159 | 2 | 1 | 20 |
| 35 | 18 | F | AR | 11.1 | 2 | 1 | 26 |
| 36 | 14 | M | BA, AR | 53.5 | 2 | 1 | 44 |

M-male; F-female; AR- Allergic rhinitis; BA- Bronchial asthma; NT-not tested; 0 - no therapy, or without infection; 1 - one dose of vaccine, or infection; 2 - two doses of vaccine; days-time from vaccination to blood collection or time from PCR-confirmed infection to blood collection

Table E3: Characterization of patients with rheumatoid diseases included in the study

| Patient no. | Age (Years) | Sex | Diagnosis/ comorbidities | DMARDs | Biologics/ corticosteroids | Other treatment | IgG (IU/ml) | IgG normal range (IU/ml) | SARS-CoV-2 vaccine | SARS-CoV-2 infection | days |
| --- | --- | --- | --- | --- | --- | --- | --- | --- | --- | --- | --- |
| 1 | 14 | M | JIA | MTX | Etanercept (TNF inh) | 0 | 14.39 | 7 - 16 | 0 | 1 | 28 |
| 2 | 13 | M | JIA | MTX | Adalimumab (anti TNF-alpha) | 0 | ND | 7 - 16 | 0 | 1 | 57 |
| 3 | 10 | F | Juvenile dermatomyositis | 0 | 0 | MTX (2018-2019), Prednison 2017 | 9.21 | 7 - 16 | 0 | 1 | 22 |
| 4 | 9 | F | JIA | MTX | 0 | 0 | 9.12 | 7 - 14 | 0 | 1 | 73 |
| 5 | 8 | M | Juvenile dermatomyositis | MTX | 0 | 0 | 10.51 | 6 - 13 | 0 | 1 | 50 |
| 6 | 7 | F | JIA | MTX | 0 | 0 | 7.44 | 6 - 13 | 0 | 1 | 82 |
| 7 | 17 | M | JIA | 0 | 0 | Adalimumab (anti TNF-alpha 2017-2018), MMF 2-7/21, MTX 2015-2018 | 8.48 | 7 - 16 | 0 | 1 | 39 |
| 8 | 9 | M | JIA | MTX | Etanercept (TNF inh) | 0 | 11.42 | 7 - 14 | 0 | 1 | 14 |
| 9 | 11 | F | JIA | MTX | 0 | Clindamycin | 12.04 | 7 - 14 | 0 | 1 | NA |
| 10 | 7 | M | JIA | MTX | 0 | 0 | 8.21 | 6 - 13 | 0 | 1 | 25 |
| 11 | 13 | F | JIA | MTX | 0 | 0 | 10.28 | 7 - 14 | 0 | 1 | 78 |
| 12 | 13 | F | JIA | MTX | Etanercept (TNF inh) | Levothyroxine | 11.01 | 7 - 16 | 2 | 0 | 80 |
| 13 | 16 | F | JIA | 0 | Adalimumab (anti TNF-alpha) | 0 | 11.01 | 7 - 16 | 2 | 0 | 35 |
| 14 | 6 | M | JIA | MTX | 0 | 0 | 8.14 | 6 - 13 | 2 | 0 | 8 |
| 15 | 15 | F | JIA | 0 | 0 | tocilizumab (anti IL-6) until 01/2021 | 11.58 | 7 - 16 | 2 | 0 | 36 |
| 16 | 14 | M | CRMO | MTX | 0 | 0 | 11.83 | 7 - 16 | 2 | 0 | 209 |
| 17 | 16 | F | JIA | MTX | Adalimumab (anti TNF-alpha) | 0 | 14.4 | 7 - 16 | 2 | 0 | 26 |
| 18 | 18 | F | Juvenile dermatomyositis | 0 |  | MTX (until6/21), CsA (2018-2019) | 9.33 | 7 - 16 | 2 | 1 | 45 |
| 19 | 12 | F | Takayasu's arteritis | 0 | Rituximab (antiCD20) | Anopyrin | 8.92 | 7 - 16 | 2 | 1 | 29 |
| 20 | 18 | M | JIA | MTX | Adalimumab (anti TNF-alpha) | 0 | 11.44 | 7 - 16 | 2 | 1 | 8 |
| 21 | 6 | F | JIA | MTX | 0 | 0 | 9.88 | 6 - 13 | 2 | 1 | 89 |

M-male; F-female; JIA- Juvenile idiopathic arthritis; CRMO- Chronic recurrent multifocal osteomyelitis; MTX- Methotrexate; DMARD- Disease-modifying antirheumatic drug; NT - not tested; IgG - Immunoglobulin class G; TNF-alpha - Tumor necrosis factor alpha; 0 - no therapy, or without infection; 1 - one dose of vaccine, or infection; 2 - two doses of vaccine; days-time from vaccination to blood collection or time from PCR-confirmed infection to blood collection

Table E4: Characterization of patients with hematopoietic stem cell transplantation included in the study

| Patient no. | Age (Years) | Sex | Primary diagnosis/ comorbidities | Year of HSCT | Current treatment/ ≤ 6 months | IgRT/ type | IgG (IU/ml) | CD3 (cell/µL) | CD19 (cell/µL) | SARS-CoV-2 vaccine | SARS-CoV-2 infection | days |
| --- | --- | --- | --- | --- | --- | --- | --- | --- | --- | --- | --- | --- |
| 1 | 19 | M | B-ALL | 2018 | Exjade, Euthyrox | 0 | 10.32 | 1.7 | 0.68 | 0 | 1 | 47 |
| 2 | 16 | M | VSAA | 2016 | 0 | 0 | 9.87 | 1.4 | 0.47 | 0 | 1 | 98 |
| 3 | 5 | F | B-ALL | 2019 | 0 | 0 | 6.13 | NT | NT | 0 | 1 | 20 |
| 4 | 11 | F | JMML | 2014 | 0 | 0 | 10.91 | NT | NT | 0 | 1 | 51 |
| 5 | 18 | F | VSAA | 2020 | Exjade, Prednison - 0,1mg/kg/day, Prograf, ATB, Sporanox, Herpesin | 0 | 5.29 | NT | NT | 2 | 0 | 97 |
| 6 | 19 | F | SAA | 2017 | Prednison 0,74mg/kg/day | 0 | 10.67 | 1.7 | 0.22 | 2 | 0 | 217 |
| 7 | 16 | M | RAEB | 2015 | 0 | 0 | 11.12 | 1.1 | 0.8 | 2 | 0 | 87 |
| 8 | 21 | M | F-HLH | 2001 | 0 | 0 | 8.96 | 1.2 | 0.26 | 2 | 0 | 124 |
| 9 | 23 | F | B-ALL | 2x - 2014, 2015 | Euthyrox, Exjade | 0 | 12.08 | 0.9 | 0.39 | 2 | 0 | 200 |
| 10 | 16 | M | B-ALL | 2x - 2015, 2016 | Euthyrox | 0 | 7.65 | 1.7 | 0.29 | 2 | 0 | 46 |
| 11 | 15 | M | RCC | 2017 | 0 | 0 | 9.02 | NT | NT | 2 | 0 | 69 |
| 12 | 24 | F | CML | 2014 | IVIG, insulins | IVIG | 4.81 | 1.9 | 0.32 | 2 | 0 | 232 |
| 13 | 19 | M | SM | 2020 | 0 | 0 | NT | NT | NT | 2 | 0 | 202 |
| 14 | 18 | F | CML | 2006 | Euthyrox, Etrofen, Duphaston | 0 | 13.57 | NT | NT | 2 | 0 | 181 |
| 15 | 19 | M | B-ALL | 2019 | Exjade | 0 | 9.22 | NT | NT | 2 | 0 | 118 |
| 16 | 14 | M | RCC | 2014 | 0 | 0 | 9.79 | NT | NT | 2 | 0 | 153 |
| 17 | 8 | F | B-ALL | 2019 | Euthyrox | 0 | 7.83 | 1.9 | 0.43 | 2 | 0 | 41 |
| 18 | 11 | M | AML | 2011 | Euthyrox | 0 | 10.04 | 1.3 | 0.45 | 2 | 0 | 21 |
| 19 | 10 | M | B-ALL | 2016 | Euthyrox | IVIG | 5.45 | 1.4 | 0.16 | 2 | 0 | 43 |
| 20 | 17 | M | B-ALL | 2010 | 0 | 0 | NT | NT | NT | 2 | 0 | 30 |
| 21 | 17 | M | ALCL | 2015 | 0 | 0 | 6.42 | 1.9 | 0.21 | 2 | 1 | 132 |
| 22 | 17 | F | B-ALL | 2010 | Euthyrox | 0 | 10.06 | 1 | 0.15 | 2 | 1 | 114 |
| 23 | 20 | M | B-ALL | 2013 | Euthyrox | 0 | 12.34 | 1.1 | 0.27 | 2 | 1 | 17 |
| 24 | 20 | M | T-ALL | 2014 | Euthyrox | 0 | 10.76 | 1.7 | 0.18 | 2 | 1 | 64 |
| 25 | 19 | M | B-ALL | 2016 | 0 | 0 | 9.3 | 1.2 | 0.21 | 2 | 1 | 57 |
| 26 | 16 | F | Fanconi anemia | 2012 | 0 | 0 | 10.36 | 1.14 | 0.19 | 2 | 1 | 98 |
| 27 | 22 | M | RCC | 2014 | 0 | 0 | 11.7 | 1.9 | 0.35 | 2 | 1 | 136 |
| 28 | 19 | M | XIAP - phenotype IBD (colitis), short stature | 2021 | CSA, corticosteroids (hydrocortisone), ATB (ciprofloxacin, co-trimoxazol), antivirotics (acyclovir), antimycotics (itrakonazol) | 0 | 10.54 | 0.9 | NT | 1 | 1 | 10 |

M-male; F-female; B-ALL- B -Cell acute lymphoblastic leukemia; VSAA- Very severe aplastic anaemia; SAA-Severe aplastic anemia; RAEB- Refractory anemia with excess blasts; F-HLH-Familial hemophagocytic lymphohistiocytosis; RCC- Renal cell carcinoma; CML- Chronic myeloid leukemia; AML- Acute myelogenous leukemia; ALCL- Anaplastic large cell lymphoma; T-ALL-T-cell acute lymphoblastic leukemia; XIAP- X-linked inhibitor of apoptosis protein deficiency; CSA- Cyclosporine A; IVIG - Intravenous immunoglobulin (Kiovig/Privigen) ; HSCT- Hematopoietic stem cell transplantation; NT-not tested; IgRT - Immunoglobulin replacement therapy; IgG - Immunoglobulin class G; 0 - no therapy, or without infection; 1 - one dose of vaccine, or infection; 2 - two doses of vaccine; days-time from vaccination to blood collection or time from PCR-confirmed infection to blood collection

TABLE E5: Titre of Spike-specific IgG, IgA and IgM antibodies

|  | **Infection** | **Vaccine** | **Hybrid** |
| --- | --- | --- | --- |
| Cohorts | **IgG - geometric mean (lower; upper 95% CI); n** | | |
| **HC** | **29623 (10687; 82113); n=8** | **209327 (117764; 372082); n=14** | **1229774 (761791; 1985000); n=8** |
| PID | 839 (7; 100040); n=8 | 153995 (46838; 506306); n=6 | 14911 (0; 1.421E+13); n=3 |
| BA-AR | 40915 (8049; 207983); n=2 | 266189 (173426; 408571); n=25 | 584314 (276387; 1235000); n=9 |
| RD | 2686 (191; 37699); n=11 | 508468 (201426; 1284000); n=6 | 147520 (25161; 864908); n=4 |
| HSCT | 26625 (7337; 96611); n=4 | 107975 (17806; 654749); n=16 | 119071 (1946; 7285000); n=8 |
| **IC** | **3323 (578; 19109); n=25** | **205023 (116074; 362136); n=53** | **172819 (33133; 901403); n=24** |
|  | **IgA - geometric mean (lower; upper 95% CI); n** | | |
| **HC** | **172 (11; 2809); n=8** | **87 (10; 726); n=14** | **21548 (6472; 71744); n=8** |
| PID | 138 (4; 5,006); n=8 | 10 (0; 424); n=6 | 933 (0; 2.296E+09); n=3 |
| BA-AR | 268 (2; 42839); n=2 | 456 (137; 1516); n=25 | 9726 (1999; 47310); n=9 |
| RD | 38 (3; 509); n=11 | 233 (2; 21981); n=6 | 278 (0; 380053); n=4 |
| HSCT | 19 (0; 5561); n=4 | 462 (43; 5003); n=16 | 2873 (111; 74653); n=8 |
| **IC** | **60 (13; 280); n=25** | **275 (97; 777); n=53** | **2672 (566; 12623); n=24** |
|  | **IgM - geometric mean (lower; upper 95% CI); n** | | |
| **HC** | **45 (1; 1386); n=8** | **9 (1; 69); n=14** | **54 (1; 1982); n=8** |
| PID | 6 (0; 95); n=8 | 1 (1; 1); n=6 | 14 (0; 1162000); n=3 |
| BA-AR | 1 (1; 1); n=2 | 2 (1; 6); n=25 | 5 (0; 45); n=9 |
| RD | 14 (1; 161); n=11 | 82 (0; 13724); n=6 | 57 (0; 96162); n=4 |
| HSCT | 1 (1; 1); n=4 | 2 (1; 7); n=16 | 34 (1; 825); n=8 |
| **IC** | **6 (2; 21); n=25** | **3 (1; 7); n=53** | **16 (3; 73); n=24** |

TABLE E6: Titre of inhibitory antibodies

|  | **Infection** | **Vaccine** | **Hybrid** |
| --- | --- | --- | --- |
|  | Inhibitory antibodies - geometric mean (lower; upper 95% CI); n | | |
| **HC** | **108 (19; 597); n=8** | **1077 (586; 1981); n=14** | **9601 (4668; 19747); n=8** |
| PID | 22 (1; 376); n=8 | 870 (163; 4645); n=6 | 485 (0; 3.321E+08); n=3 |
| BA-AR | 246 (1; 84909); n=2 | 1693 (1207; 2375); n=25 | 5769 (2099; 15857); n=9 |
| RD | 19 (3; 132); n=11 | 2778 (1411; 5469); n=6 | 1210 (116; 12603); n=4 |
| HSCT | 9 (0; 475); n=4 | 665 (150; 2949); n=16 | 1556 (94; 25663); n=8 |
| **IC** | **22 (7; 71); n=25** | **1252 (770; 2036); n=53** | **2108 (658; 6760); n=24** |

TABLE E7: CD4 T-cells response

|  | **Infection** | **Vaccine** | **Hybrid** |
| --- | --- | --- | --- |
|  | CD4 T-cells - geometric mean (lower; upper 95% CI); n | | |
| **HC** | **35 (8; 158); n=5** | **20 (10; 39); n=12** | **37 (14; 99); n=8** |
| PID | 8 (3; 27); n=8 | 27 (4; 189); n=6 | 12 (0; 3387); n=3 |
| BA-AR | 38 (23; 62); n=2 | 20 (12; 35); n=24 | 20 (8; 50); n=8 |
| RD | 17 (6; 46); n=11 | 70 (13; 373); n=6 | 29 (4; 215); n=4 |
| HSCT | 16 (3; 77); n=4 | 18 (9; 35); n=16 | 17 (5; 58); n=8 |
| **IC** | **14 (8; 25); n=25** | **23 (16; 35); n=52** | **19 (11; 34); n=23** |


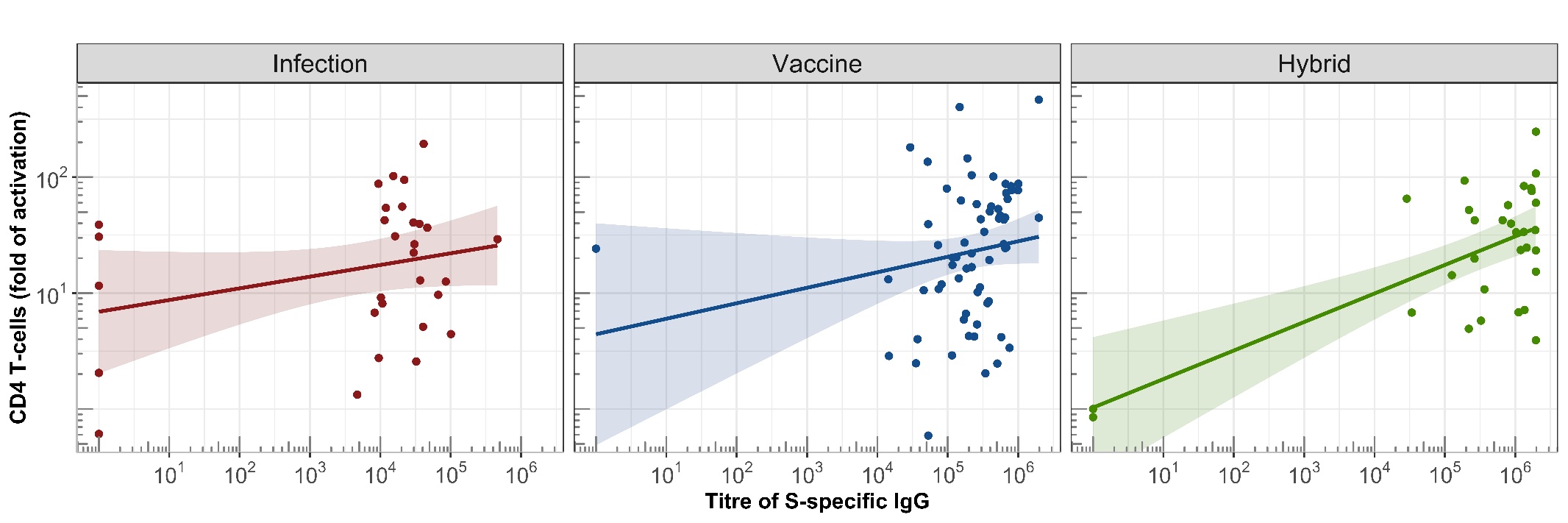


FIGURE E1. Correlations between titers of Spike-specific IgG and CD4 T-cells (fold of activation) for pooled immunocompromised and healthy cohorts. The linear regression line together with 95% confidence bands for a regression line in log-scale are visualized for each group. Spearman’s correlation coefficient (r_s_ ) was calculated to assess the association between the titre of S-specific IgG and CD4 T-cells, supplemented with statistical significance level based on p-value; infection: r_s_=0.167, p=0.35713; vaccine: r_s_=0.333, p=0.00565; hybrid: r_s_=0.341, p=0.05611.
